# Development of novel biliary metal stent with coil-spring structure and its application *in vivo* swine biliary stricture model

**DOI:** 10.3389/fonc.2023.1103217

**Published:** 2023-02-13

**Authors:** In Rae Cho, Sang Hyub Lee, Jin Ho Choi, Namyoung Park, Min Woo Lee, Joo Seong Kim, Seok Jeong, Don Haeng Lee, Tae-Won Jeong, Byoung-Yun Ki, Woo Hyun Paik, Ji Kon Ryu, Yong-Tae Kim

**Affiliations:** ^1^ Department of Internal Medicine and Liver Research Institute, Seoul National University College of Medicine, Seoul, Republic of Korea; ^2^ Department of Gastroenterology, Kyung Hee University Hospital at Gangdong, Seoul, Republic of Korea; ^3^ Department of Internal Medicine, Dongguk University College of Medicine, Dongguk University Ilsan Hospital, Goyang-si, Republic of Korea; ^4^ Digestive Disease Center, Department of Internal Medicine, Inha University School of Medicine, Incheon, Republic of Korea; ^5^ The National Center of Efficacy Evaluation for the Development of Health Products Targeting Digestive Disorders (NCEED) and Utah-Inha DDS & Advanced Therapeutics Research Center, Incheon, Republic of Korea; ^6^ CG Bio Co., Ltd., Seoul, Republic of Korea

**Keywords:** endoscopic retrograde cholangiopancreatography, biliary tract neoplasms, bile duct obstruction, stent, animal experiment

## Abstract

**Background:**

As of date, endoscopic biliary stenting with plastic stent (PS) and self-expandable metal stent (SEMS) have been widely used for the palliation of biliary tract strictures. However, these two stents have several limitations regarding the management of biliary strictures caused by intrahepatic and hilar cholangiocarcinoma. PS has short patency and also risks bile duct injury and bowel perforation. SEMS is difficult to revise when occluded by tumor overgrowth. To compensate for such shortcomings, we developed a novel biliary metal stent with coil-spring structure. The aim of this study was to investigate the feasibility and efficacy of the novel stent in a swine model.

**Methods:**

The biliary stricture model was prepared in six mini-pigs using endobiliary radiofrequency ablation. Conventional PS (n=2) and novel stents (n=4) were deployed endoscopically. Technical success was defined as successful stent placement and clinical success was defined as >50% reduction of serum bilirubin level. Adverse events, stent migration, and endoscopic removability for one month after stenting were also assessed.

**Results:**

The biliary stricture was successfully created in all animals. The technical success rate was 100 %, and the clinical success rate was 50% in the PS group and 75% in the novel stent group. In the novel stent group, the median pre- and post-treatment serum bilirubin levels were 3.94 and 0.3 mg/dL. Stent migration occurred in two pigs and two stents were removed by endoscopy. There was no stent-related mortality.

**Conclusions:**

The newly designed biliary metal stent was feasible and effective in a swine biliary stricture model. Further studies are needed to verify the usefulness of the novel stent in the management of biliary strictures.

## Introduction

Biliary stricture can be caused by various diseases and the majority of biliary strictures are malignant (1, 2). Although endoscopic biliary stenting is considered as the preferred treatment modality, the management of malignant hilar and intrahepatic duct strictures remains a clinical challenge. The lengthy distance from the duodenal papilla to the stricture site requires a long stent (generally >10 cm) and severe angulation of the bile duct causes difficulties in stent deployment. Also, the thin diameter of the intrahepatic bile duct and the presence of branch ducts make it technically more challenging than extrahepatic strictures. Thus, it is difficult to define the optimal drainage strategy and stent type for the palliation of hilar and intrahepatic duct stricture.

As of date, two types of stents are conventionally used in clinical practice - plastic stent (PS) and self-expandable metal stent (SEMS) (3). However, PS and SEMS still have several limitations to overcome in the management of hilar and intrahepatic strictures. PS has an advantage in terms of cost and convenience in deployment and exchange (4). However, PS shows a relatively short patency due to clogging caused by biofilm formation and therefore requires frequent exchange (5, 6). The axial force of PS may also increase the risk of complications such as stent dislocation and intestinal perforation (7). SEMS, with larger caliber about 10mm, is generally considered to have a longer patency than PS. However, the uncovered SEMS (ucSEMS) cannot be removed, and revision is difficult when obstructions from tumor progression occur (8). Fully covered SEMS (fcSEMS) can be removed if required and provides longer stent patency by preventing tumor ingrowth. Nevertheless, it has the risk of migration and associated risks of intrahepatic bile duct branch occlusion and subsequent cholangitis and abscess formation (9).

With the improvement of pancreatobiliary cancer prognosis due to advances in chemotherapy (10–12), there is a growing clinical need for a novel stent to overcome the limitations of PS and SEMS and also effectively manage malignant hilar and intrahepatic biliary stricture. Thus, we devised a novel biliary metal stent with the following characteristics: larger inner diameter compared to PS with same outer diameter, more flexibility due to coil-spring structure, and easily removable. Using the novel biliary metal stent, we performed a three-stage preclinical study.

The aim of this study is to verify the validity of the novel biliary metal stent with coil-spring structure and investigate its feasibility and efficacy in a swine biliary stricture model.

## Materials and methods

### Novel biliary metal stent with coil-spring structure, delivery system and phantom model

**Figure 1:**
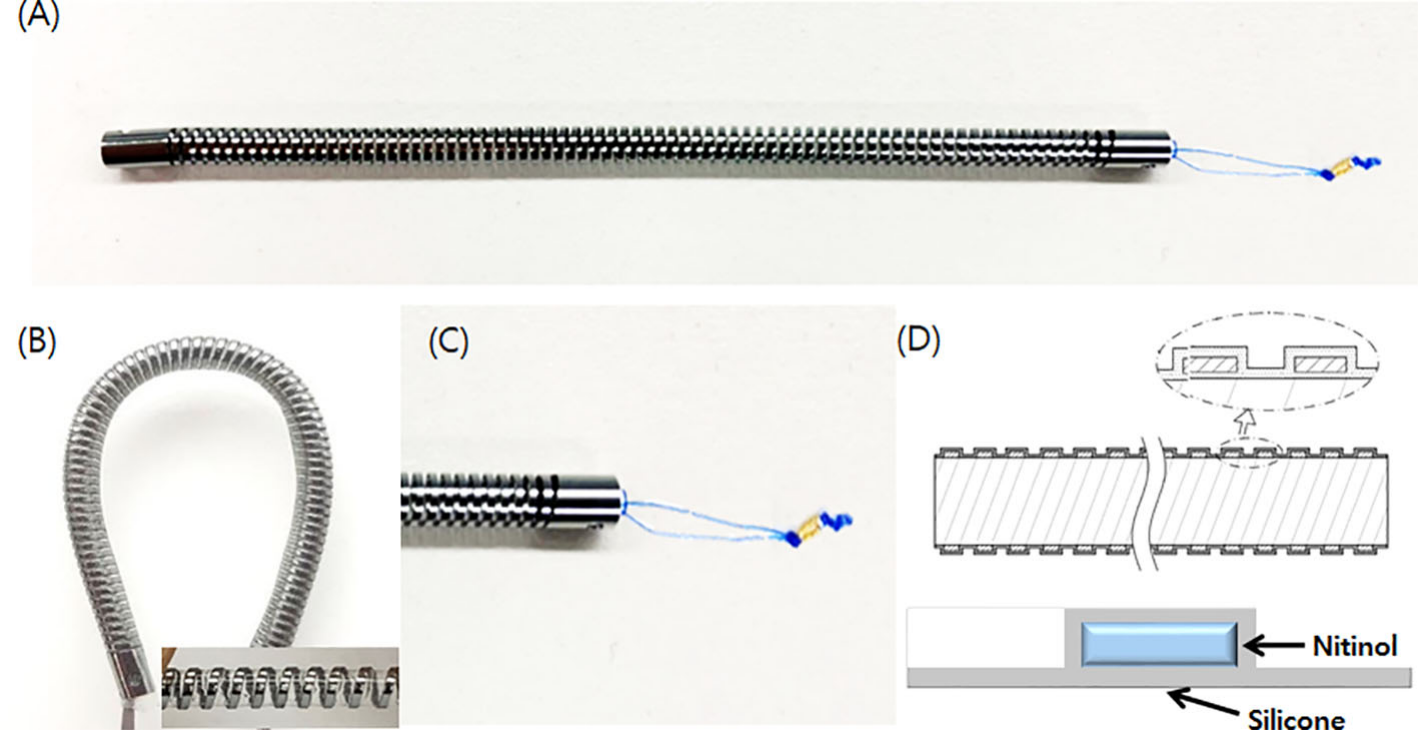
Novel biliary metal stent with coil-spring structure. **(A)** Structure of novel stent. **(B)** Coil-spring structure was adopted to increase flexibility and prevent migration. **(C)** A silicone skirt was installed at the distal end to prevent duodenal mucosa damage and a lasso was attached for endoscopic removal. **(D)** Silicone coating was applied to both sides of the nitinol.

In this preclinical study, we used the novel biliary metal stent (as experimental) and conventional PS (as control). The structure of the novel biliary metal stent with coil-spring structure (CG Bio Co., Ltd., Seoul, Korea) is shown in **Figure 1**.** **A metal stent was processed into a coil-spring structure through laser cutting. The metal material used was nitinol and silicone membrane was coated inside and outside the stent. By adopting the coil-spring structure, the novel stent was designed to increase flexibility and have high compliance with the bile duct structure. To prevent migration, there were irregularities on the outside of the stent. Silicone skirt and a lasso were attached to the distal end to prevent migration and enable easy removal. The Cotton-Leung^®^ Biliary Stent (Cook Medical, Bloomington, IN, USA) with 8.5 Fr outer diameter and 7 cm length was used as conventional PS.

The novel biliary metal stent had the following features: 400mg weight, 8.5 Fr outer diameter, and 7 cm length. Compared to a conventional PS with the same outer diameter, the novel stent had 23.8% larger cross-sectional area inside the lumen (diameter: 2.6mm vs. 2.1mm). In addition, flexibility and resistance to external compression force increased by 92.8% and 92.1%, respectively, a result of the 3-p bending test. Stents are delivered with a 9-Fr pull-back delivery system, which is compatible with the working channel of a duodenoscope. It is designed for easy access to the biliary stricture site with high flexibility and pushability.

**Figure 2:**
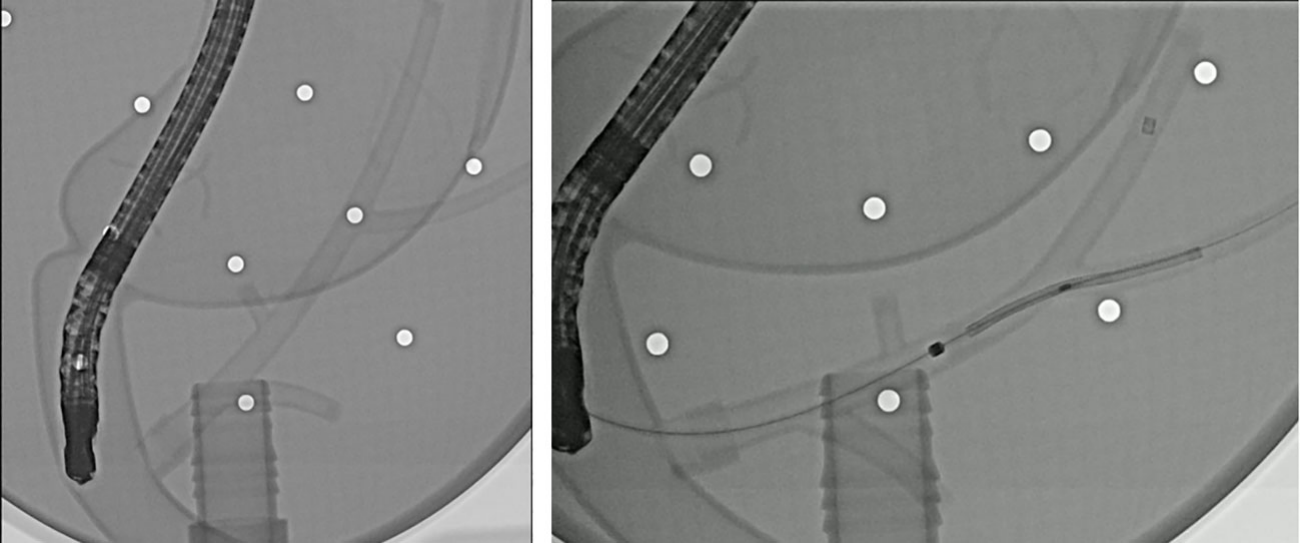
Fluoroscopic images of the endoscopic deployment of a novel biliary metal stent in a phantom model.

To verify the validity of the stent and delivery system, usability test was performed using the phantom model (Medical training tools, Roosendaal, Netherlands) made like the human body. Through the test, we could confirm that the stent was deployed without any problems and adapted well to the biliary structure (**Figure 2**).

### Animals

A total of six mini-pigs (*Sus scrofa*; Medi Kinetics Co. Ltd., Pyeongtaek, Korea) were used for the *in vivo* experimental study (**Supplementary Figure 1**). The mean age of the mini-pigs were 14 months and each subject weighed approximately 30 kg. All animals were quarantined and acclimated in a vivarium for one week before the experiments. They were kept in specific pathogen-free facilities with complete substrate feeding. All animals fasted overnight and were given only water for 24 hours before the endoscopic procedure. Pre-anesthesia sedation consisted of an intramuscular injection of tiletamine/zolazepam (5mg/kg), xylazine hydrochloride (2mg/kg), and atropine sulfate (0.04 mg/kg). The animals were subsequently intubated, and general anesthesia was achieved with 1.5% isoflurane (Foran^®^; JW Pharmaceutical Corp., South Korea). During the procedure, heart rate, respiratory rate, and oxygen saturation were monitored continuously. The pigs resumed their usual diet (1000 kcal/day) on the day after the procedure.

### Ethical statement

This animal study was approved by the Institutional Animal Care and Use Committee at the KNOTUS Co. Ltd, (IRB No. KNOTUS IACUC 21-KE-025). All procedures carried out for the animal experiment were performed in accordance with the ARRIVE guidelines (13). The introduction and breeding of animals were carried out according to the institutional ethics guidelines and ARRIVE guidelines, under the supervision of KNOTUS employees. No unnecessary animal discomfort was caused during the experiment including the endoscopic procedure. All authors complied with the ARRIVE guidelines, and the outcome measures and statistical analysis also followed the guidelines.

### Endoscopic procedure for making biliary stricture model

**Figure 3:**
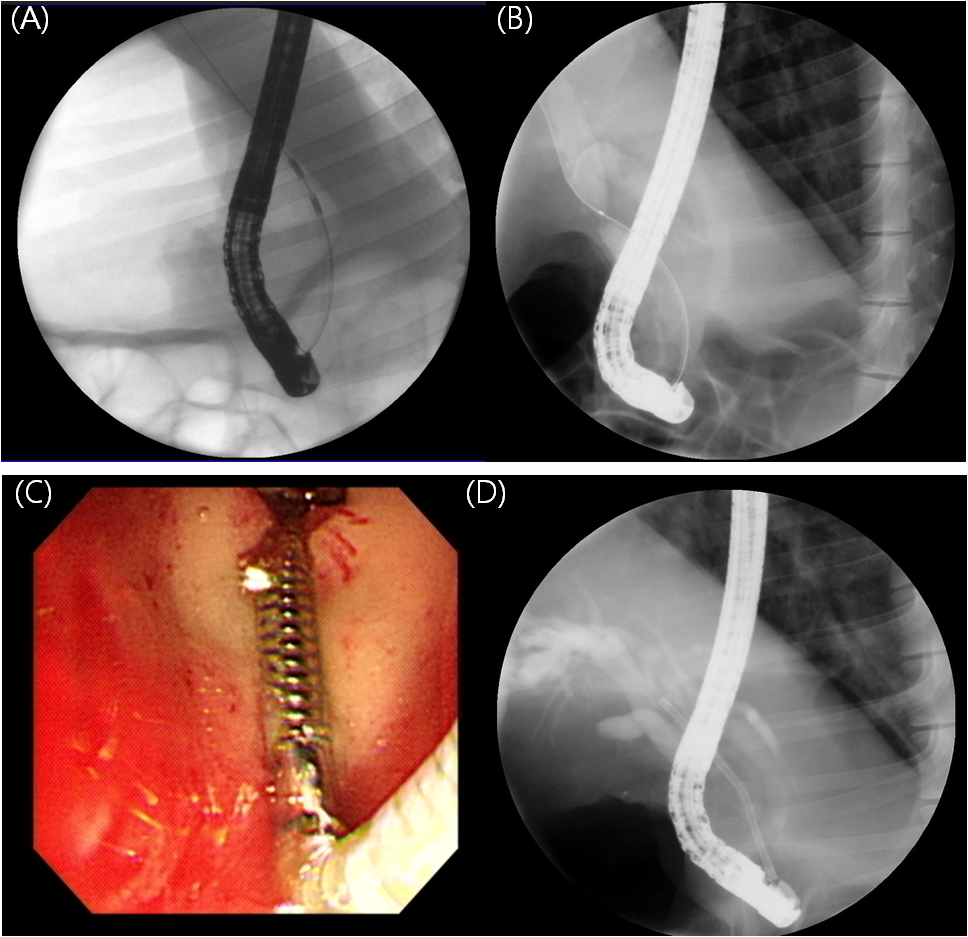
Endoscopic retrograde cholangiograms of animals. **(A)** Cholangiogram obtained during endobiliary RFA. RFA catheter positioned in the CBD **(B)** Cholangiogram at four weeks after endobiliary RFA showing stricture at site of procedure **(C)** Novel biliary metal stent was deployed successfully. Distal tip located in the duodenal bulb. **(D)** Stent was deployed across the stricture site.

Endoscopic procedures were performed by experienced endoscopists (I.R.C. and S.H.L.) using a standard side-viewing duodenoscope (TJF-240; Olympus Optical Co. Ltd., Tokyo, Japan) under general anesthesia. After the endoscope was inserted into the duodenum, the bile duct orifice was identified. A 0.035-inch hydrophilic-tipped guidewire (Cook Medical, Bloomington, IN, USA) was inserted into the bile duct using the pull-type sphincterotome (MicroTech, Nanking, China). After obtaining the cholangiogram, endoscopic sphincterectomy was performed and the endobiliary radiofrequency ablation (RFA) catheter (18mm electrode length, 7F diameter, 175-cm working length; STARmed Co., Ltd, Goyang, Korea) was advanced into the common bile duct (CBD) over the guidewire under fluoroscopic guidance. RFA applications (VIVA combo RF generator, VCS10, STARmed Co., Ltd) were performed at an energy setting of 7W, 80° C for 80 seconds according to the previously published protocol (**Figure 3**) (14).

During the 4 weeks following the initial procedure, the animals were fed their usual diet. Clinical signs and parameters including weight loss, daily food intake, and demeanor score were monitored daily and X-rays and biochemical laboratory tests were assessed every week.

### Stent deployment

After 4 weeks of the RFA procedure, the second endoscopic procedure was performed to deploy a stent. Novel coil-spring stents were deployed in four pigs (experimental group) while conventional PS were inserted into the two pigs (control group). First, the duodenoscope was inserted into the duodenum, and the cholangiogram was obtained. After confirming the biliary stricture was well created, the novel metal stent or PS was inserted over a guide wire using a delivery system under fluoroscopic guidance and deployed across the stricture site (**Figure 3**). Technical success was defined as successful stent placement in an appropriate position (across the stricture) without any immediate adverse events.

### Follow−up and clinical parameters

**Figure 4:**
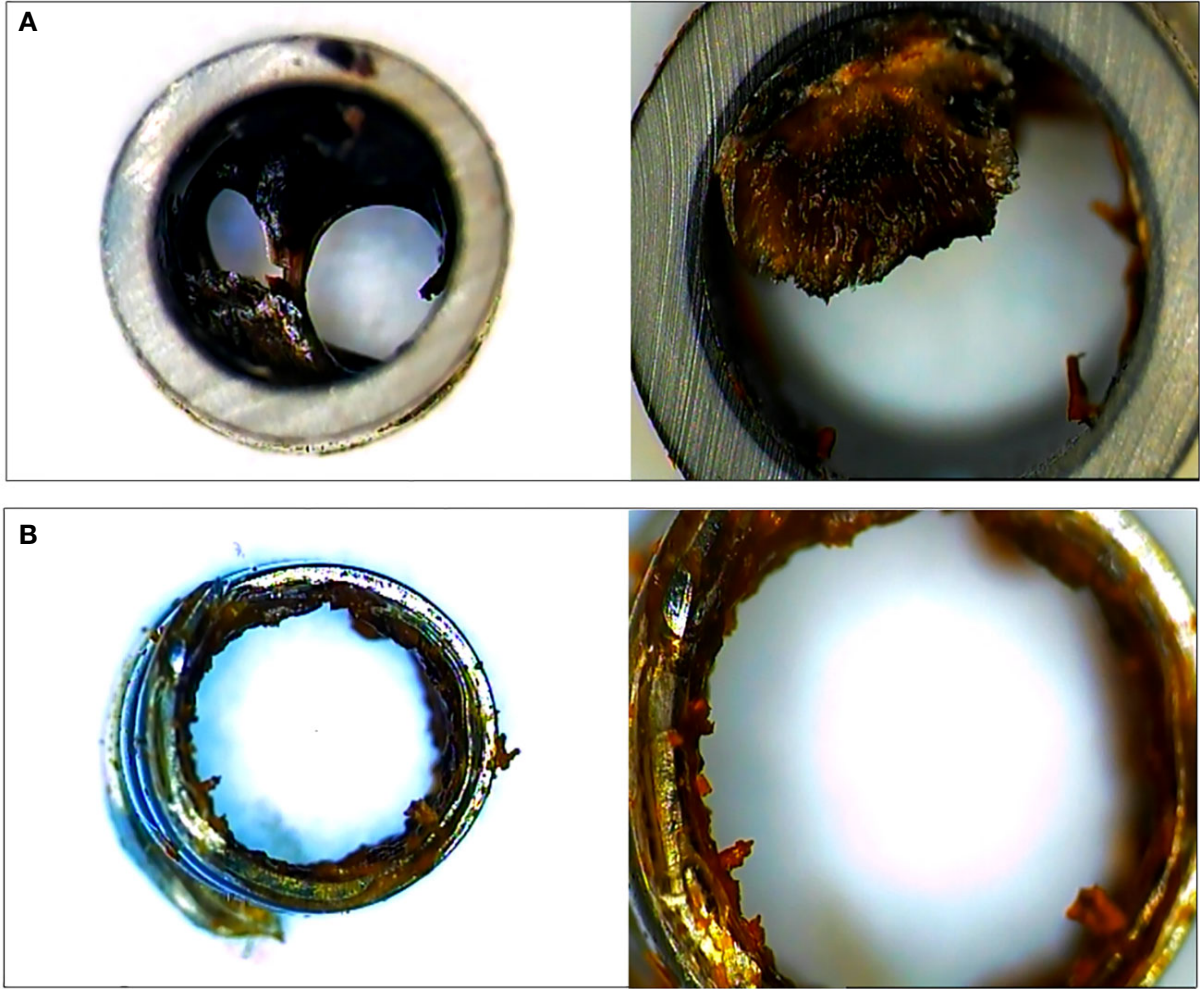
The cross section of plastic stent **(A)** and novel biliary metal stent **(B)** were checked after endoscopic stent removal. Both stents had been placed for 4 weeks. Novel biliary metal stent showed a large inner diameter through less formation of biofilm and biliary sludge.

During 4 weeks after stent insertion, the animals were fed their usual diet and clinical evaluation was performed daily to confirm improvements in jaundice and detect any adverse events (AEs). X-ray and biochemical laboratory tests were assessed every week. Clinical success was defined as a reduction in serum bilirubin level of >50% during the first week after stent placement. For other clinical parameters, the stent patency and migration were evaluated. Regarding stents that were placed for more than 4 weeks, endoscopic removal using rat tooth forcep was performed, and follow-up cholangiogram was taken one week after stent removal (or migration) and necropsy was performed. All the retrieved stents were cut and the cross section was investigated to check the changes in the lumen such as biofilm formation or sludge clogging (**Figure 4**).

### Histologic examination

**Figure 5:**
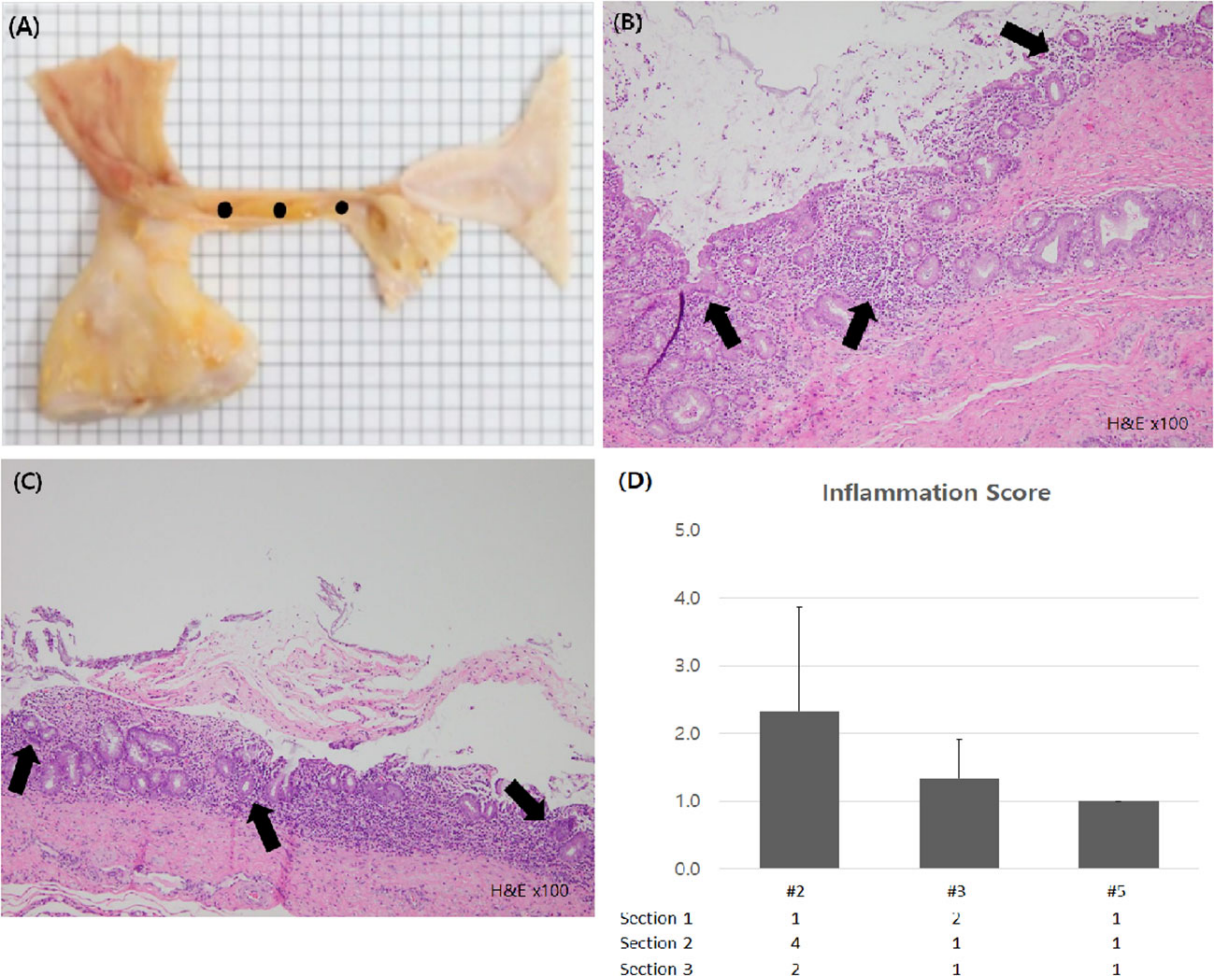
Histologic examination of bile duct. **(A)** Gross inspection of extracted bile duct was conducted. For microscopic examination, fixed sample was sectioned at 3 levels (black dots). **(B, C)** Inflammatory cell infiltration sites were seen in the bile duct mucosa of subject #2 and #3 (black arrow, H&E X100) **(D)** Inflammation scores of each subject were calculated and compared.

After the experiment, all animals were euthanized and assessed for bleeding, perforation or damage to surrounding structures in peritoneal cavity. After removing the stent endoscopically, the bile duct was excised with a longitudinal incision. The excised bile duct was grossly observed, and then, the formalin-fixed bile duct samples were cross-sectioned at three levels for microscopic examination: the distal and proximal ends, and the middle of the stented segment (**Figure 5A**). The sectioned tissue samples were stained with hematoxylin-eosin and examined by an experienced pathologist (T-W. J.) who evaluated the degree of submucosal inflammatory cell infiltration. The degree of submucosal inflammatory cell infiltration was graded as 0 (none), 1 (mild), 2 (moderate), or 3 (severe).

### Statistical analysis

All statistical analyses were performed using SPSS for Windows version 23.0 (IBM, Armonk, NY, USA). Categorical variables were described as subject number and proportions for all subjects. Continuous variables were described as medians and interquartile ranges.

## Results

### Endobiliary RFA procedure and technical aspects

Endobiliary RFA procedure was successfully performed on all subjects. There were no technical difficulties or immediate adverse events during the procedure. On the follow-up fluoroscopy 4 weeks after the procedure, biliary stricture was observed in all subjects and there was no difference in the degree of stricture between the control and experimental groups (**Supplementary Figure 2**). The novel biliary metal stent was deployed in four subjects and PS was deployed in two subjects. Technical success rate was 100 % (6/6), and no procedure-related complications were encountered during stent deployment. Also, there was no difficulty when removing the stents. The novel coil-spring stent could be easily removed through the endoscopy working channel by holding the lasso with a rat tooth forcep.

### Clinical parameters and follow−up in the animal model

The treatment details and outcomes of all subjects are summarized in **Table 1**. Among the control group subjects, one pig (subject #1) expired due to severe cholangitis eight days after the procedure. In other subjects (#2) in the control group, a 78.3% decrease in serum bilirubin level was confirmed after the procedure, and the stent was placed for seven weeks without migration.

**Table 1:** Characteristics of all subjects that underwent endoscopic stent deployment.

Subject No	Group	Technical success	Clinical success	Stent migration	Indwelling period (days)	Endoscopic removal	T.bil reduction (%)
1*	Control	Yes	No	.	8	.	.
2	Control	Yes	Yes	No	42	Success	78.30%
3	Experimental	Yes	Yes	No	35	Success	89.00%
4	Experimental	Yes	No	Yes	28	.	46.70%
5	Experimental	Yes	Yes	No	35	Success	91.90%
6	Experimental	Yes	Yes	Yes	21	.	54.20%

*Subject was expired due to severe cholangitis.

In the novel stent group, three of four (75%) subjects showed clinical success. Stent migration occurred in two subjects and two stents were removed successfully by endoscopy five weeks after the deployment. The median stent indwelling period was 31.5 days. Stent migration was confirmed by plain X-ray, and there was no bowel injury due to stent migration. Adverse events affecting the survival of the subjects did not occur. **Table 2** showed pre- and post-treatment laboratory values of novel stent group subjects. After stent deployment, median white blood cell count (24,490/mm^3^ to 21,920/mm^3^), aspartate aminotransferase (AST, 81.2 U/L to 51.7 U/L), and total bilirubin level (3.94 to 0.3 mg/dL) were reduced.

**Table 2:** Changes in laboratory values of the novel stent group subjects.

	Pre-Treatment	Post-Treatment
WBC, mm^3^	24,490 (14,292-35,522)	21,920 (14,450-31,765)
T.bilirubin, mg/dL	3.94 (0.92-6.23)	0.3 (0.18-2.52)
AST, U/mL	81.2 (65.9-205.4)	57.1 (35.1-389.0)
ALT, U/mL	23.1 (21.5-27.2)	24.6 (19.6-31.5)

Values expressed as median and interquartile range.

### Luminal change evaluation of retrieved stents and histologic examination of bile duct

To evaluate luminal patency, the cross sections of the endoscopically retrieved stents (one plastic stent and two novel biliary metal stents) were investigated. In comparison to PS, the novel biliary metal stent was confirmed with less biofilm formation and sludge clogging in the stent lumen (**Figure 4**).

Through histologic examination, we found that the inflammation score of the novel stent group was lower than that of the PS group. The inflammation score of subject #2, in which PS was deployed and removed endoscopically, was 2.3 ( ± 1.5), whereas in subjects #3 and #5, in which the novel biliary metal stent was inserted and removed, the scores were 1.3 (± 0.6) and 1.0 (± 0.0), respectively (**Figure 5**). There was no evidence of intra-abdominal bleeding, perforation, or bowel damage in any of the subjects in autopsy.

## Discussion

This preclinical *in vivo* study shows that the novel biliary metal stent with coil-spring structure can be a potentially effective instrument for biliary stricture. The technical success rate was 100 % and clinical success was achieved in most subjects. The novel metal stent could be easily removed endoscopically and did not induce severe inflammation in the bile duct epithelial tissue. In addition, there were no adverse events related with the procedure and after self-migration of the stent. Although there are limitations in the relatively short length of the stent and the biliary stricture swine model, we established a basis for the development of a novel biliary metal stent. This novel coil-spring metal stent with improved stent structure, has the potential to serve as an efficient alternative for the palliation of hilar and intrahepatic duct strictures.

Unlike extrahepatic duct stricture, the successful endoscopic management of intrahepatic and perihilar duct stricture caused by intrahepatic and hilar cholangiocarcinoma are very difficult to achieve (15). There are technical difficulties and the risk of post-procedural cholangitis due to stricture complexity and the possibility of incomplete drainage (16–18). Both PS and SEMS have their pros and cons, so the optimal strategy for these types of biliary strictures remains unclear. Although ucSEMS is superior to PS in many aspects, it has disadvantages including impossible stent exchanges and difficult revision (19). In particular, recently, local therapy such as photodynamic therapy and RFA have been performed for biliary tract cancer patients who are not eligible for operation, and PS is more desirable in this case due to requirements for stent replacement (20, 21). In addition, for the treatment of anastomotic stricture after liver transplantation, repeated stent exchange using PS has been performed, and thus replaceable stents are also more useful in such cases (22). Therefore, a novel biliary metal stent with coil-spring structure which is flexible, exchangeable, and has prolonged patency could play an important role.

The novel biliary metal stent has potential advantages derived from its unique structure (**Supplementary Table 1**). Compared to PS, which has limitations in enlarging the outer diameter beyond the size of the duodenoscope working channel, the novel metal stent has a larger inner diameter and effective cross-sectional area due to less sludge clogging and biofilm formation. Also, the novel stent has no side hole which could prevent the generation of microturbulence that occurs around the side hole and causes sludge formation (23). In addition, it has a coil-spring structure that can respond to external forces in a more flexible manner. Therefore, even with stent migration, the risk of causing bowel obstruction or perforation is relatively low. In comparison to ucSEMS, the novel stent has advantages in terms of multiple stenting and revision. Thus, it can be useful for the palliation of proximal bile duct stricture. In addition, a smaller diameter may facilitate bilateral, multiple stent insertion, and possibly prevent branch bile duct occlusion that may occur in fcSEMS. Based on such properties, this novel biliary metal stent could be useful for patients who require multiple stent insertion in the proximal bile ducts, repeated local therapies such as RFA, and long-term placement and exchange of stents.

A number of challenges need to be solved through follow-up studies after this preclinical study. Most of the existing swine biliary stricture models were established in a way that induces benign stricture of the extrahepatic biliary duct (14, 24). We also created biliary stenosis in the extrahepatic bile duct due to difficulties in establishing an appropriate and reproducible intrahepatic bile duct stricture model. Therefore, the efficacy and safety of a ‘long’ coil-spring stent for intrahepatic bile duct drainage could not be fully evaluated. Further research is necessary to evaluate the usefulness of a long stent through a hilar stricture model. In addition, stent migration occurred in 50% of subjects in the novel stent group. As flared ends could cause bile duct injury and embedment (25), the novel biliary metal stent was structured without flared ends and an anti-migration feature was attempted through surface irregularities. This is another unsolved problem that needs structural improvement and further preclinical research. Structural improvements such as the adoption of a pigtail structure or the application of anti-migration flaps are needed. However, despite such concerns, the novel biliary metal stent with coil-spring structure displayed potentials of safety and clinical usability. No adverse events such as bowel perforation or impaction were observed even after migration, and no duodenal ulceration was reported after more than 4 weeks of deployment. It was confirmed to induce less inflammation of the bile duct epithelium compared to PS. In addition, clinical success was confirmed even in the subjects that showed early migration.

We learned several points from this preclinical study. In the technical aspect, endoscopic procedures for mini-pigs were more difficult than for humans. The stomach of mini-pig is larger than that of a human and has more acute angulation (26). Also, the location of the ampullary orifice was different, (just below the pyloric channel) and the bowel was more redundant. Therefore, the duodenoscope conventionally used for humans was relatively short in length. The difficulty increased further as the swine continued to grow during the experiment. Thus, the experimental period was not sufficient to evaluate stent migration and patency with endoscopy. It is thought that stent migration and patency can be investigated through follow-up of X-ray and laboratory value for a sufficient period without additional endoscopic procedure. The absence of antibiotics administration raised concerns about ascending cholangitis after the procedure and the contrast medium was not sufficiently injected. So, the configuration of the proximal bile duct and cystic duct could not be sufficiently evaluated in some subjects. Therefore, there is a possibility that the stent was deployed in inappropriate position that could be related with modest clinical success and stent migration. It is necessary to make a more precise protocol in the future. Biliary stricture created by RFA-induced inflammation has a benign nature and can influence pathological evaluation after stent removal. Efforts are needed to find methods to discriminate the RFA-induced inflammation and additionally induced inflammations by stent insertion. Based on the lessons mentioned above, we believe that we can design a more delicate plan in follow-up experiments, and hope that the lessons of this study will be helpful to other researchers who are planning similar experiments.

The present study has several limitations. First, the sample size was too small to confirm the clinical effect and compare the efficacy and safety between stents. We aimed to confirm the feasibility and safety of the novel stent through this study, and thus the sample size was not sufficient to prove the clinical benefits of the novel stent. Although there was no additional inflammation and less sludge formation in the novel stent group compared to the control group, the small sample size makes it difficult to generalize. Second, this study was conducted using only the extrahepatic duct stricture model using RFA which cannot fully reflect the characteristics of malignant stricture. Therefore, it was impossible to check the effect of the common causes of stent failure in malignant biliary strictures such as tumor overgrowth or ingrowth to the novel stent patency. Further study is needed after establishing an intrahepatic bile duct or malignant stricture model based on the experience of this study. Third, stents with the same length, diameter and shape were used. The novel stent was in its early stage of development and therefore the novel stent used in the study had relatively short length and similar outer diameter to the existing PS. Finally, there were limitations in demonstrating clinical success with limited number of subjects and a relatively short period of study. Some subjects in experimental group showed lower bilirubin reduction rate due to stent migration and persistent cholangitis. Improvement of stent structures for anti-migration and the appropriate use of antibiotics should be accompanied to improve clinical outcomes.

In conclusion, the novel biliary metal stent with coil-spring structure was feasible and effective in the *in vivo* swine biliary stricture model. Furthermore, novel biliary metal stent removal was successful after a one-month stent-indwelling period. Therefore, the novel biliary metal stent may facilitate biliary stricture treatment. Further long term studies with a larger number of subjects in various locations of the biliary stricture are needed to verify the usefulness of the novel stent.

## Data availability statement

The original contributions presented in the study are included in the article/**Supplementary Material**. Further inquiries can be directed to the corresponding author.

## Ethics statement

The animal study was reviewed and approved by Institutional Animal Care and Use Committee at the KNOTUS Co. Ltd.

## Author contributions

IC contributed to the data collection and interpretation, performance of endoscopies, and manuscript draft writing. SL contributed to the study conception and design, performing endoscopy, and study supervision. JC, NP, ML, JK contributed to the performing endoscopies and data collection and interpretation. SJ and DL contributed to the study conception and interpret the endoscopic findings. T-WJ contributed to the tissue preparation, histological examination and interpretation. B-YK contributed to the stent development and study conception and design. WP, JR, Y-TK contributed to the data interpretation and manuscript draft writing. All authors contributed to the article and approved the submitted version.
