# Supplementary material for: Development of novel biliary metal stent with coil-spring structure and its application in vivo swine biliary stricture model
Source: Front Oncol. 2023 Feb 13;13:1103217. doi: 10.3389/fonc.2023.1103217 (PMC9982730; doi:10.3389/fonc.2023.1103217)
Supplement: Supplementary file 1 [file Table_1.docx]

| Supplemental Table 1. Comparisons between the novel metal stent, plastic stent and self-expandable metallic stents | | | | |
| --- | --- | --- | --- | --- |
|  | Novel metal stent^§^ | Plastic stent^§^ | uc-SEMS | fc-SEMS |
| Diameter | 2.79 mm (Inner: 2.60 mm) | 2.74 mm (Inner: 2.10 mm) | 10 mm | 10 mm |
| Patency | Longer than PS^*^ | Short (about 3 mo.) | Long (about 6 mo.) | Long (about 6 mo.),  if not dislocated |
| External force required to reduce the diameter by 1.37 mm | 106.4N | 55.4N | N/A | |
| External force required to reduce the diameter by 2mm | 1.056N | 0.076N | N/A | |
| Exchange | Possible | Possible | Impossible | Possible |
| Multiple stenting | Possible | Possible | Very difficult | Difficult |
| Tumor ingrowth | Rare^*^ | Rare | More | Less |
| Migration | Probable^*^ | Possible | Impossible | Probable |
| Cost | Cheaper than SEMS^*^ | Cheap | Expensive | Expensive |
| § 8.5Fr of outer diameter * expected property (not yet used conventionally) Abbreviations: uc-SEMS, uncovered self-expandable metal stent; fc-SEMS, fully covered self-expandable metal stent, N/A; not applicable | | | | |
