# Supplementary figures and images for: Development of novel biliary metal stent with coil-spring structure and its application in vivo swine biliary stricture model
Source: Front Oncol. 2023 Feb 13;13:1103217. doi: 10.3389/fonc.2023.1103217 (PMC9982730; doi:10.3389/fonc.2023.1103217)

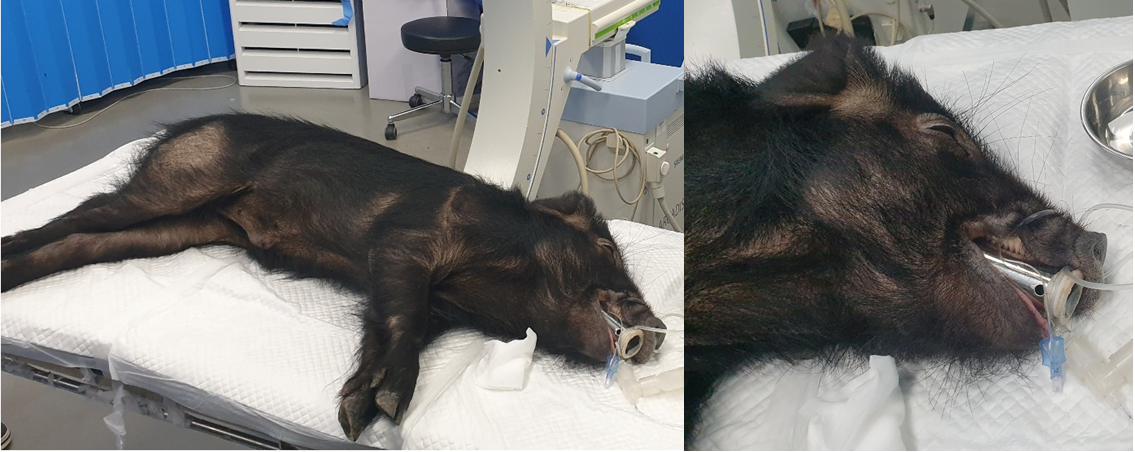

Supplement: Supplementary file 2 [file Image_1.tif]

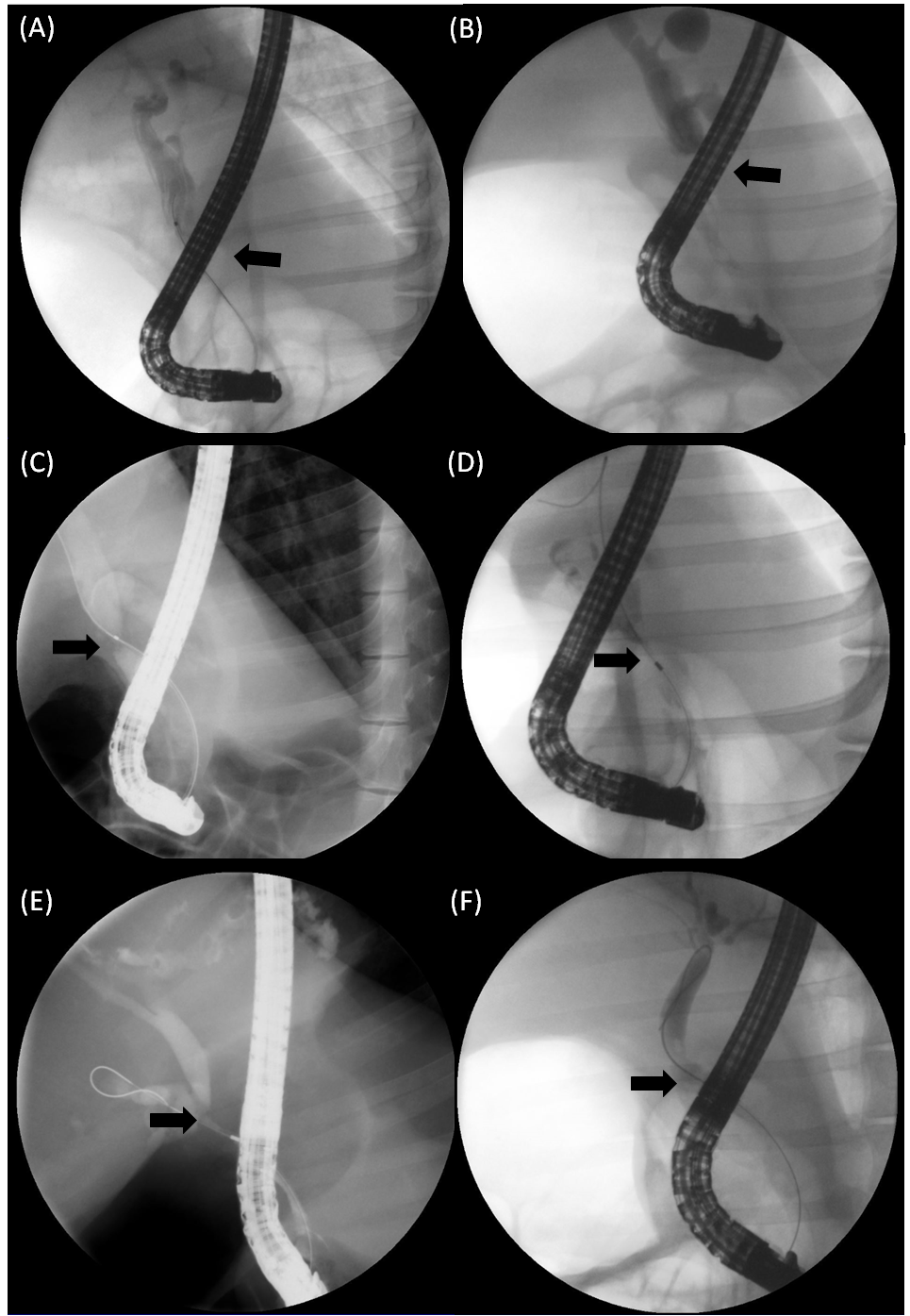

Supplement: Supplementary file 3 [file Image_2.tif]
